# Subphrenic Abscess in Appendicitis

**Published:** 1903-02-14

**Authors:** 


### SUBPHRENIC ABSCESS IN APPENDICITIS.

In a study of the records of 4,028 autopsies collected from the Boston City Hospital, 1896 to 1902, the Johns Hopkins Hospital, 1889 to 1902, and the Rhode Island Hospital, 1900 to 1902, made by Dr. Henry A. Christian, of Boston,[1] and Dr. Louis C. Lehr, of Baltimore, it was found that death was due directly or indirectly to acute appendicitis in 86. Of these 86 cases seven, or 8·13 per cent., showed an involvement of the subphrenic region in a purulent process, and though in a strict sense of the word not each one of these was a case of subphrenic abscess, yet the figures illustrate the comparative frequency with which this condition occurs as a complication secondary to appendicitis. The origin of subphrenic abscesses is very various. Those on the right side commonly arise by direct extension of an inflammatory process from the liver, from appendicular and periappendicular lesions, from perforation of the duodenum and more rarely of the stomach; while on the left side perforation of the stomach is the most frequent cause. These abscesses may contain only purulent material, or this may be mixed with gas, the latter either coming from perforation of some air-containing viscus, or being produced *in situ* by the bacterial flora present—the *Bacillus aerogenes capsulatus* or some one of the *Bacillus coli communis* group. From a consideration of figures collected from various sources it would seem that subphrenic abscess is not a very unusual complication of appendicitis. In the seven cases described by the authors the affection was unilateral six times—four times on the right and twice on the left—and in one case it was bilateral. Subphrenic abscess secondary to appendicitis mostly arises they say by extension of the diseased process from the appendix by an intraperitoneal route, or by an extension outside the peritoneum by way of the lymphatics, or by infiltration through the retroperitoneal tissues. Which route is taken will depend much on the situation of the appendix and the periappendicular process. If the latter be retrocæcal and so extraperitoneal the chances are that the process will take an extraperitoneal route reaching the subphrenic region by way of the loose areolar tissue in the lumbar region. On reaching the subphrenic region extraperitoneally the abscess may remain so or may become intraperitoneal. The record of these cases is interesting, since it may, and doubtless sometimes does, happen that a case of subphrenic abscess, the origin of which may seem undiscoverable, may be the result of a previous attack of appendicitis.

[1] Vol. xiii, Medical and Surgical Reports of the Boston City Hospital.

